# Large skin defect in Type V aplasia cutis congenita treated with conservative treatment: a case report

**DOI:** 10.1186/s12887-024-04777-0

**Published:** 2024-05-07

**Authors:** Yan song, Ru yang, Zeyao shi, Jing yan, Shulin hou, Xiaowen li, Xiufang zhao

**Affiliations:** 1 Department of Neonatology Nursing, West China Second University Hospital, Sichuan University, Chengdu, China; 2 Department of Nursing, West China Second University Hospital, Sichuan University, Chengdu, China; 3 Key Laboratory of Birth Defects and Related Diseases of Women and Children, (Sichuan University), Ministry of Education, Chengdu, China

**Keywords:** Type V aplasia cutis congenital, Conservative treatment, Hydrogel foam, Silicone foam dressings

## Abstract

Aplasia cutis congenita (ACC) is a congenital disorder that can be classified into nine types, with Type I ACC being the most common. Type V ACC associated with fetus papyraceus is a rare subtype of ACC. We report the case of a Type V ACC in a male newborn with extensive abdominal skin defects. The patient received conservative treatment using hydrogel foam and silicone foam dressings. Approximately five weeks later, the patient was discharged when more than 60% of the skin had completed epithelialization. After discharge from West China Second University Hospital, Chengdu , the patient continued to be followed up regularly at the Burns and Plastic Surgery Clinic at local hospital in Gansu. We followed up the child by telephone. After 4 months of follow-up, scar tissue formation was observed in the trunk area. The infant is 2 years and 5 months old now, physical examination did not reveal any organ problems.

## Introduction

Aplasia cutis congenita (ACC) is a rare, congenital disorder characterized by localized or widespread absence of skin at birth with various etiologies and heterogeneous clinical presentation [1]. The precise incidence of ACC is unknown but thought to be around 0.5/10 000 in Europe [2]. However, the actual incidence may be higher, because cases of mild ACC are probably underreported [3]. Skin lesions can occur anywhere on the body, but 80% and even more cases of ACC have involved the scalp, others (15%) could be found on the trunk and limbs [2, 4]. ACC may be an isolated skin malformation involving one or more skin districts, and it may also merge with multiple other organ malformations [5]. The defect may be superficial, depth varies from the absence of epidermis and upper dermis only to subcutaneous tissue. In some severe cases, it may extend to deeper tissues, such as the muscle or bone (periosteum, skull, and dura).

The most widely used classification of ACC was proposed by Frieden in 1986. Frieden grouped ACC into nine subtypes based on its associated abnormalities, inheritance pattern, and body area affected [6]. Type I ACC is the most common type, and most of them are without underlying syndromes, malformations, or other abnormalities. Type V is rare, associated with fetus papyraceus or placental infarction, and characterized by a symmetrical and H-shaped distribution over the trunk and extremities [7]. Here, we successfully treated a patient with extensive abdominal skin defects with conservative treatment.

## Case history

**Fig. 1:**
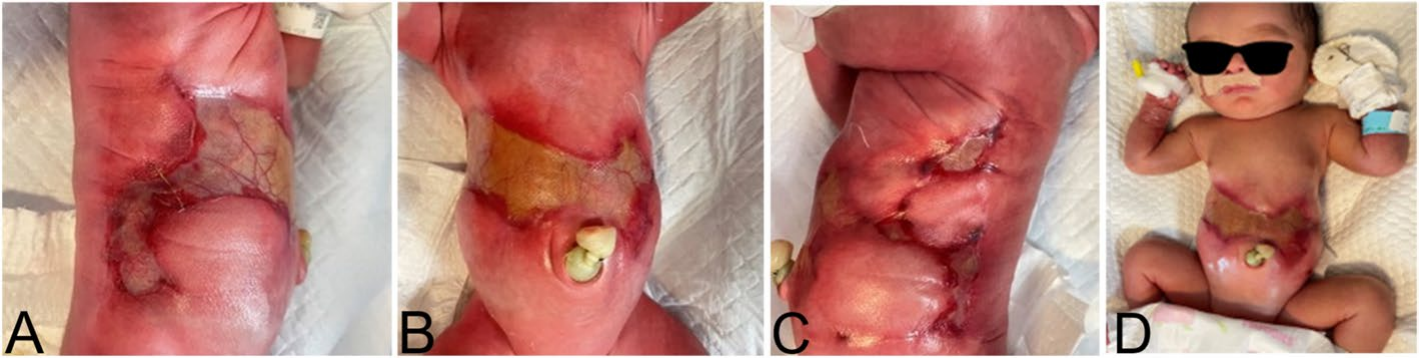
Symmetrical skin defects in the abdomen and bilateral trunk

An infant with a birth weight of 2350g, was delivered through cesarean section at 37 weeks of gestation at a local hospital in Gansu (438 km far from West China Second University Hospital, Chengdu). The mother was a 35-year-old healthy primigravid woman who conceived a monocorionic diamniotic twin pregnancy naturally. The mother experienced fetal demise of one fetus at 13-14 weeks of gestation. During the pregnancy, the mother had no history of taking medicine and did not experience remarkable complications during the rest of the pregnancy. The surviving one was admitted to our hospital less than 24 hours after birth due to significant skin defects on the trunk. There were symmetrical skin defects in the abdomen and bilateral trunk, measuring approximately 17 × 4 cm. The lesions appeared to be a complete absence of epidermis, dermis, and subcutaneous fat. The defects exhibited clear boundaries and were covered only by a thin gelatinous translucent membrane, revealing visible ribs and blood vessels (Fig. 1). No skin defects were observed in other areas, and the remaining physical examination and complementary exams such as ultrasound of the chest and abdomen showed no abnormality. These indicated that the patient has no combination of deformities or defects other than abdominal skin defects.

**Fig. 2:**
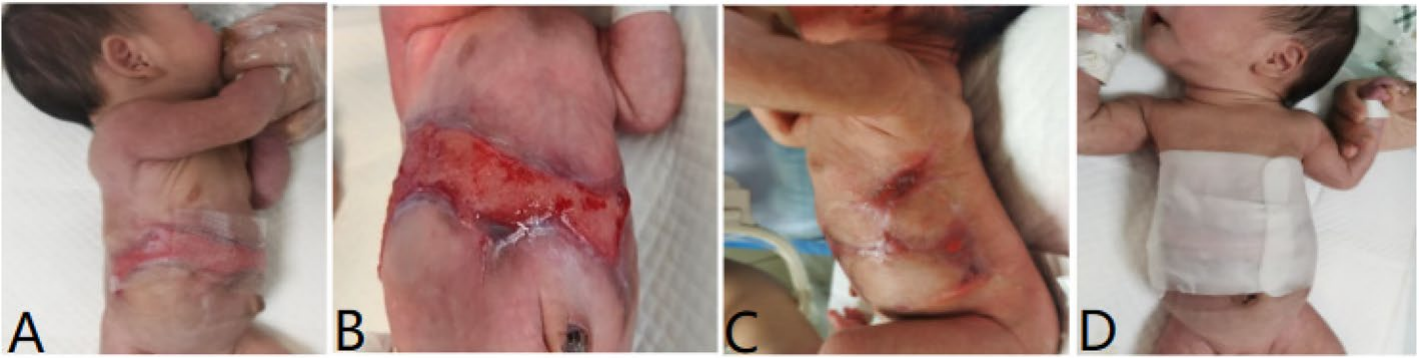
Significant improvement after 5 weeks with lipid hydrogel foam dressing, silicone foam dressing, and growth factors

**Fig. 3:**
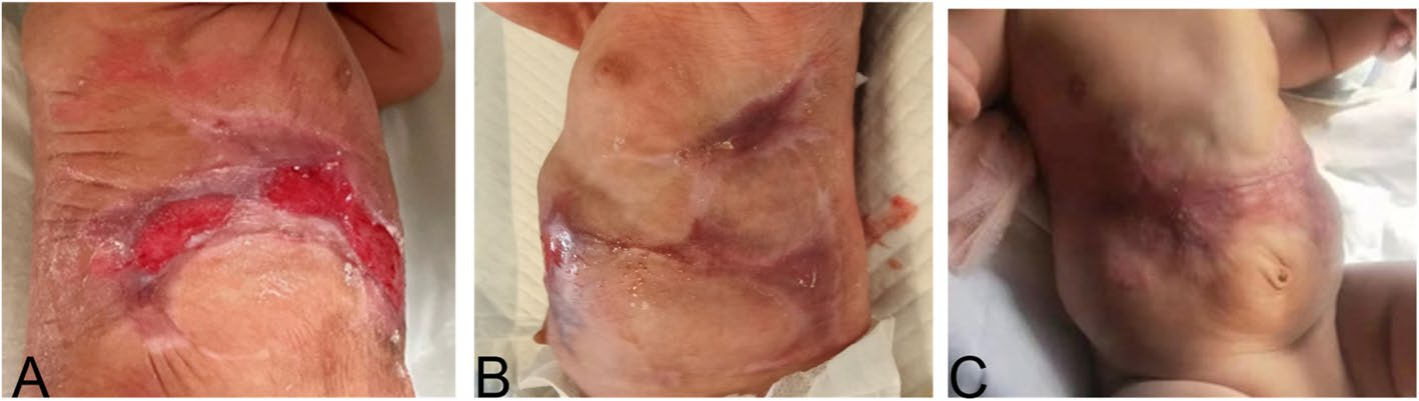
More than 60% of the lesions showed epithelialization (**A**, **B**), and scar formation was observed in the trunk area after 4 months (**C**)

The patient was diagnosed with "Type V aplasia cutis congenita" based on the clinical manifestations and the Frieden classification. Due to the large defect skin and the presence of vital organs in the abdominal area such as the liver, and intestines. The primary focus of the patient was the local wound management. The main principles of wound treatment included maintaining adequate moisture in the wound, promoting epithelial tissue growth, and preventing infection. The patient received treatment with lipid hydrogel foam dressing (Urgotul®), silicone foam dressing (ALLEVYN GENTLE BORDER), and growth factors (Fig. 2). Additionally, prophylactic antibiotics were administered intravenously using Cefoperazone sodium tazobactam sodium. Following approximately five weeks of the treatment, the patient was discharged from the neonatal intensive care unit (NICU) when more than 60% of the lesion showed epithelialization. Throughout the hospitalization period, the patient did not experience systemic or local infections, nor did they develop severe complications such as electrolyte imbalance or bleeding. After discharge from the West China Second University Hospital, Chengdu, the patient continued to be followed up regularly at the Burns and Plastic Surgery Clinic for dressing changes and observation of wound healing at local hospital in Gansu. We followed up the child by telephone. After 4 months of follow-up, scar tissue formation was observed in the trunk area (Fig. 3). The infant is 2 years and 5 months old now, physical examination did not reveal any organ problems.

## Discussion and conclusions

Type V ACC is rare, associated with fetus papyraceus or placental infarction, and characterized by a symmetrical and H-shaped distribution over the trunk and extremities. The pathogenesis of ACC is unknown, multiple factors might be contributing to the development of ACC. ACC is associated with chromosomal abnormalities, amniotic irregularities, vascular disorders, fetus papyraceus, and pregnancy infection (herpes simplex virus and varicella virus). Otherwise, the use of some teratogenic drugs during the mother’s pregnancy might increase the risk of ACC, such as misoprostol, benzodiazepines, valproic acid, cocaine, and methimazole [8]. Besides, several genes are causing Adams-Oliver Syndrome (diagnosed in the presence of ACC of the scalp and terminal transverse limb defects), such as DLL4, NOTCH1, DOCK6 and EOGT [9, 10]. Several heterozygous mutations in COL7A1 may result in Bart’s syndrome, which is characterized by ACC and epidermolysis bullosa (EB) [11, 12]. Genetic mutations in the COL7A1 gene are also associated with dystrophic EB [13]. Moreover, ACC can be associated with physical defects or syndromes. In the case of children diagnosed with trisomy 18, membrane ACC has been observed on the skull [14, 15].

Several theories have been proposed to explain the pathomechanism of Type V ACC. Although the exact cause is not yet known, most recent evidence tended to support transient hypovolemia as the primary cause [16]. Intrauterine death of one twin results in rapid blood shunting from the surviving fetus to the dying one [17]. Especially in monochorionic pregnancies, this acute transfusion between twins could induce acute hypovolemia in the living twin, resulting in ischemia of the skin and other organs [18, 19]. Monochorionic twin pregnancies have greater morbidity risk than dichorionic twin pregnancies, possibly because placental vascular anastomoses produce anomalous fetal-fetal transfusion [20]. Arteriovenous vascular anastomoses are found in 90-95% of monochorionic placentas [21]. Otherwise, studies suggested that in the presence of late first to early second-trimester fetal death in an initial twin gestation pregnancy, small abdominal circumference, detectable acetylcholinesterase, high amniotic and maternal alpha-fetoprotein could suggest the diagnosis of ACC with fetus papyraceous [22].

Most ACCs were generally treated with conservative measures and took approximately 6 weeks to fully epithelialize. Conservative treatment focuses on keeping the wound sufficiently moist, promoting epithelial tissue growth, and preventing infection [23]. Conservative treatment includes various dressings, topical antimicrobials, and systemic antibiotics [24]. Dressings used include ionic silver dressing, moist exposed burn ointment, hydrogels, adhesive or nonadherent dressings, basic fibroblast growth factor, and recombinant human epidermal growth factor [25–28]. The benefit of conservative treatment of ACC is the avoidance of the risks associated with surgery. However, when there is a large area of skin defect in important areas, such as the scalp, surgical approaches can be used to prevent morbidity and mortality from central nervous system related complications such as meningitis, sagittal sinus bleeding, and cerebral hernia. Surgical options include local or free flaps, skin grafting, acellular dermal matrix, allogenic dermis, and cultured epithelial allografts [29–35]. A child with a large total body surface area of ACC (37%) involving the entire scalp, chest, and trunk was successfully treated using acellular dermal matrix and cultured epithelial allografts [32]. Other surgical treatments of ACC on the scalp or skull include delayed cranioplasty, early composite cranioplasty, and tissue expanders [36].

The patient received conservative treatment with lipid hydrogel foam dressing (Urgotul®), silicone foam dressing (ALLEVYN GENTLE BORDER), and growth factors. Following approximately five weeks of the treatment, the patient was discharged from the NICU when more than 60% of the lesion showed epithelialization. Most children with Type V ACC can be treated conservatively with satisfactory results.

## Data Availability

No datasets were generated or analysed during the current study.

## Abbreviations

ACC: Aplasia cutis congenita
NICU: Neonatal intensive care unit
EB: Epidermolysis bullosa
